# A biochemical feedback signal for hypothermia treatment for neonatal hypoxic–ischemic encephalopathy: focusing on central nervous system proteins in biofluids

**DOI:** 10.3389/fped.2024.1288853

**Published:** 2024-05-03

**Authors:** Hongyan Lv, Qiuli Wang, Fang Liu, Linhong Jin, Pengshun Ren, Lianxiang Li

**Affiliations:** ^1^ Department of Neonatology, Handan Maternal and Child Health Care Hospital, Handan, Hebei, China; ^2^ Department of Neonatal Pathology, Handan Maternal and Child Health Care Hospital, Handan, Hebei, China; ^3^ Department of Pediatrics, The 980^th^ Hospital of the PLA Joint Logistics Support Force, Shijiazhuang, China

**Keywords:** hypothermia, neonatal hypoxic-ischemic encephalopathy, central nervous system-specific proteins, biomarker, biochemical signals, tau protein, neurogranin

## Abstract

Hypothermia has been widely used to treat moderate to severe neonatal hypoxic–ischemic encephalopathy (HIE), yet evaluating the effects of hypothermia relies on clinical neurology, neuroimaging, amplitude-integrated electroencephalography, and follow-up data on patient outcomes. Biomarkers of brain injury have been considered for estimating the effects of hypothermia. Proteins specific to the central nervous system (CNS) are components of nervous tissue, and once the CNS is damaged, these proteins are released into biofluids (cerebrospinal fluid, blood, urine, tears, saliva), and they can be used as markers of brain damage. Clinical reports have shown that CNS-specific marker proteins (CNSPs) were early expressed in biofluids after brain damage and formed unique biochemical profiles. As a result, these markers may serve as an indicator for screening brain injury in infants, monitoring disease progression, identifying damage region of brain, and assessing the efficacy of neuroprotective measures. In clinical work, we have found that there are few reports on using CNSPs as biological signals in hypothermia for neonatal HIE. The aim of this article is to review the classification, origin, biochemical composition, and physiological function of CNSPs with changes in their expression levels after hypothermia for neonatal HIE. Hopefully, this review will improve the awareness of CNSPs among pediatricians, and encourage future studies exploring the mechanisms behind the effects of hypothermia on these CNSPs, in order to reduce the adverse outcome of neonatal HIE.

## Introduction

Neonatal hypoxic–ischemic encephalopathy (HIE) is a relatively common cause of death and disability in newborns. The incidence of neonatal HIE in full-term infants ranges from 1 to 2 per 1,000 infants (1). In countries with higher prevalence of poverty, its incidence was 10–20 per 1,000 live births (2); in these patients, approximately 15%–20% of affected newborns die during the perinatal period. In addition, 25% of patients were showed to have neurological disability during childhood (3–6), which causes a heavy burden to families and society. In recent years, preclinical and clinical trials have shown that hypothermia have positive protective effects (7, 8), and is a safe and effective treatment method for neonatal HIE (9, 10). However, hypothermia also has some potential side effects, such as clotting disease, arrhythmia, and parental emotional stress (11, 12). At present, the effects assessment of neuroprotective hypothermia for neonatal HIE mainly relied on clinical signs and symptoms, neuroimaging (magnetic resonance imaging or computed tomography), amplitude-integration electroencephalography (aEEG), and follow-up outcomes data of patient. These methods have their own limitations. For example, MRI requires transport of the neonate to the MRI suite and generally takes approximately 40–45 min, which is not appropriate for unstable neonates. Hypothermia may depress the aEEG and thus limit its ability for early outcome prediction (13). Therefore, there is an important clinical value using biomarkers to estimate the effects of hypothermia, biomarkers can be used to support early diagnosis and differential diagnosis for neonates HIE. A few hours after brain damage, central nervous system (CNS)-specific proteins (CNSPs) can pass the endothelial tight junctions in the blood-brain barrier (BBB), and migrate into biofluids, and these biomarkers can quickly be detected. Due to difference locations and functions of these biomarkers, their expression levels can reflect different sites and degrees of brain damage. Once these CNSPs enter cerebrospinal fluid (CSF), blood, urine, saliva, tears, amniotic fluid, or other biofluids, they can considered as markers of brain damage. Therefore, CNSPs are used as biochemical signal, and reflected the effects of hypothermia for neonatal HIE.

## Neuroprotective effects of hypothermia for neonatal HIE

Hypothermia for neonatal HIE first appeared in the 1950s. Miller et al. and Westin et al. found that rapidly reducing the body temperature to 23°C–32°C could effectively reduce neonatal mortality and neurological sequelae. In 1958, Silverman et al. reported that hypothermia could increase neonatal mortality, leading to widespread abandonment of hypothermia treatment for neonatal HIE. In 1998, Gunn et al. first reported selective brain hypothermia for infants with perinatal asphyxia, with safe and effective results. From then on, therapeutic hypothermia again attracted the attention of scholars and gradually used in clinical practice. Nevertheless, the neuroprotective mechanisms of hypothermia remain unclear. It was reported that the effects of hypothermia are related to reducing energy metabolism and improving brain tissue health and function (14, 15), inhibiting free radicals (16), reducing the release of excitatory amino acids (17), inhibiting the inflammatory response (18), preventing neuronal apoptosis (19), and promoting regeneration of nerve cells (20).

## Classification of CNSPs

Biomarkers are biochemical indicators that can label the structural or functional changes of organs, tissues, cells, and ultra-structure, they have the characteristics of objectively evaluating the physiological status, disease diagnosis, the severity of disease, predicting disease outcome, evaluating the safety and effectiveness of interventions (new drugs or new therapies). Biomarkers are divided into proteins, sugars, nucleotides, which come from cell synthesis, metabolism, and some components of themselves.

CNSPs are products of CNS cells that can be released at specific time points or during certain disease statuses. CNSPs are found in the CSF and can be transmitted into blood or other biofluids (including saliva, urine, tears, amniotic fluid, etc.). The expression levels of CNSPs can reflect the pathological changes and severity of brain disease as well as side effects and adverse outcomes of hypothermia for neonatal encephalopathy. Clinically, biomarkers from brain injury can be detected early, and have important clinical value for diagnosing disease, evaluating treatment effects, making treatment plans, and predicting outcomes. Moreover, biomarkers have been received increasing attention in pediatrics and have been showed to be highly valuable (21). So far, few clinical studies investigated the effect of hypothermia for neonatal HIE on biochemical signals, including neuron-specific enolase (NSE), myelin basic protein(MBP), S100B, glial fibrillary acidic protein(GFAP), ubiquitin carboxyterminal hydrolase-L1(*UCHL1*), Tau protein, brain-derived neurotrophic factor(BDNF), phosphorylated axonal neurofilament heavy chain **(***PNF-H***)** and Neurogranin (Ng). The detailed data are summarized in Table 1.

**Table 1:** Effect of hypothermia for neonatal HIE on the central nervous specific protein markers.

Author/reference	Case (*n*)	Hypothermia model	Temperature and time	Sample/method	Outcome
NSE					
Sun et al. (22)	23	SHC	NPT: 34°C ± 0.2°C RT: 35°C Duration: 72 h	CSF (ELISA)	Hypothermia group: 27.2 ± 9.8 ug/L < control group: 36.6 ± 14.1 ug/L, *p* < 0.05
Sun et al. (23)	47	SHC	NPT: 33.5°C–34°C RT: 34.5°C–35°C Duration: 72 h	Serum (RI)	At 24 h after treatment: treatment group (23.26 ± 2.89) > control group (24.41 ± 5.91), *p* > 0.05 At 48 h after treatment: treatment group (38.12 ± 7.96) > control group (48.67 ± 9.96), *p* < 0.05 At 72 h after treatment: treatment group (31.9 ± 10.14) > control group (41.78 ± 3.46), *p* < 0.05
Jia et al. (24)	63	SHC	RT: 34°C–35°C ST: 33°C–34.5°C AFT: 20°C–25°C Duration: 72 h	Serum (ELISA)	Hypothermia group (<6 h, time window): 29.5 ± 27; (≥6 h, time window) 41 ± 25.6 control group (<6 h, time window): 25.6 ± 10.9; (≥6 h, time window) 38.1 ± 24.6 comparison between the two groups: *p* < 0.01
Alshweki et al. (25)	31	WBH	ET: 33.5°C Duration: 72 h	Serum (ELISA)	Unfavorable outcome group: Day 1 (Hypothermia): 109.1 ± 4.92 ng/ml Day 2 (Hypothermia): 113.5 ± 59.4 ng/ml Day 3 (Hypothermia): 97.3 ± 59.8 ng/ml Favorable outcome group: Day 1 (Hypothermia): 84.3 ± 28.8 ng/ml Day 2 (Hypothermia): 68.6 ± 20.6 ng/ml Day 3 (Hypothermia): 54.1 ± 16.3 ng/ml Unfavorable outcome group > Favorable outcome group, *p* < 0.05
Leon-Lózano et al. (26)	53	NDD	RT: 34°C–35°C Duration: 72 h	CSF (ACI)	Moderate or severe HIE: Electrical seizures cases during hypothermia treatment: 133 ng/ml No electrical seizures cases during hypothermia treatment: 28 ng/ml Moderate or severe injury; 133 ng/ml Global injury pattern (MRI): 166 ng/ml No global injury pattern (MRI): 29 ng/ml Cerebray palsy: 268 ng/ml
MBP					
Wang et al. (27)	49	SHC	NPT: 33.5°C–34°C Duration: 72 h	Serum (ELISA)	After 3 days of hypothermia treatment: Serum MBP level of moderate HIE was 5.94 ± 0.68 ug/L, which was significantly lower than of before treatment 7.02 ± 0.53 ug/L (*P* < 0.05); Serum MBP level of severe HIE was 6.55 ± 0.74 ug/L, which was significantly lower than of before treatment 8.30 ± 0.73 ug/L (*P* < 0.05).
S100β					
Sun et al. (22)	23	SHC	NPT: 34°C ± 0.2°C RT: 35°C Duration: 72 h	CSF (ELISA)	Hypothermia group: 0.81 ± 0.32 ug/L < control group: 1.92 ± 0.39 ug/L, *p* < 0.05
Alshweki et al. (25)	31	WBH	ET: 33.5°C Duration: 72 h	Serum (ELISA)	Unfavorable outcome group (serum): Day 1 (Hypothermia): 3.71 ± 5.21 ug/L Day 2 (Hypothermia):1.84 ± 3.88 ug/L Day 3 (Hypothermia): 1.21 ± 2.14 ug/L Favorable outcome group (serum): Day 1 (Hypothermia): 1.77 ± 2.01 ug/L Day 2 (Hypothermia): 0.86 ± 1.31 ug/L Day 3 (Hypothermia): 0.46 ± 0.38 ug/L Unfavorable outcome group > favorable outcome group, *p* < 0.05
Alshweki et al. (25)	31	WBH	ET: 33.5°C Duration: 72 h	Urine (ELISA)	Unfavorable outcome group (Urine): Day 1 (Hypothermia): 10.58 ± 14.82 ug/L Day 2 (Hypothermia): 5.16 ± 7.63 ug/L Day 3 (Hypothermia): 1.81 ± 3.60 ug/L Favorable outcome group (serum): Day 1 (Hypothermia): 4.55 ± 9.16 ug/L Day 2 (Hypothermia): 0.83 ± 2.53 ug/L Day 3 (Hypothermia): 0.46 ± 0.93 ug/L Unfavorable outcome group > favorable outcome group, *p* < 0.05
GFAP					
Chalak et al. (28)	20	WBH	ET: 33.5°C Duration: 72 h	Serum (ELISA)	At 6–24 h after treatment (moderate/severe HIE): 0.06 (0.03–0.15) pg/ml At 48–72 h after treatment: 0.05 (0.03–0.14) pg/ml At 78–96 h after treatment: 0.08 (0.04–0.20) pg/ml
Jiang et al. (29)	31	WBH	RT: 34°C Duration: 72 h	Serum (ELISA)	At 6–12 h after treatment (moderate/severe HIE) 0.090 ± 0.005 ng/ml > before treatment (moderate/severe HIE) 0.020 ± 0.007 ng/ml, *p* < 0.05
Dietrick et al. (30)	155	NDD	NDD	Plasma or CSF (ELISA)	Moderate to severe cerebral injury: Plasma GFAP level: 251 pg/ml pg/ml (9–1,154 pg/ml); CSF GFAP level: 247 pg/ml (156–415 pg/ml) Cognitive abnormal or death: Plasma GFAP level: 284.6 pg/ml (8.7–821.8 pg/ml) Abnormal motor: Plasma GFAP level 429.1 pg/ml (8.7–1,039.1 pg/ml) Abnormal language: Plasma GFAP level 99.1 pg/ml (8.7–902.6 pg/ml) * In plasma, GFAP level was directly associated with encephalopathy grade
Li et al. (31)	62	WBH	ET: 33.5°C Duration: 72 h	Plasma (ELISA)	The relationships between plasma GFAP level and MRI has not significant.
Tau protein					
Lv et al. (32)	41	SHC	ET: 33.5°C–34°C Duration: 72 h	Serum (ELISA)	At 24 h before treatment (alone Hypothermia group): 891.56 ± 221.70 pg/ml. After hypothermia treatment: At 4th day (867.59 ± 198.99 pg/ml) At 8th day (756.58 ± 203.49 pg/ml) At 12th day (697.68 ± 168.41 pg/ml) At 8th day and At 12th day after treatment < At 24 h before treatment, *p* < 0.05. At 24 h before treatment (EPO + Hypothermia Group): 913.381 ± 217.46 pg/ml. After alone hypothermia treatment: At 4th day (895.73 ± 220.69 pg/ml) At 8th day (601.22 ± 187.35 pg/ml) At 12th day (517.68 ± 143.47 pg/ml) At 8th day and At 12th day after treatment < At 24 h before treatment, *p* < 0.05.
Dietrick et al. (30)	155	NDD	NDD	Plasma or CSF (ELISA)	Moderate to severe cerebral injury: Plasma Tau level: 276.7 pg/ml (13.5–554.4 pg/ml); CSF Tau level: 2,626.1 pg/ml (56.6–6,721.5 pg/ml) Cognitive abnormal or death: Plasma Tau level 284.6 pg/ml (210.8–1,410.2 pg/ml) Abnormal motor: Plasma Tau level 412.8 pg/ml (210.8–1,410.2 pg/ml) Abnormal language: Plasma Tau level 412.8 pg/ml (209.1–1,410.2 pg/ml) * In plasma, Tau level was directly associated with encephalopathy grade and MRI.
McGowan et al. (33)	103	WBH	ET:33.5°C Duration: 72 h	Plasma/ELISA	Tau was the strongest predictor of adverse neurodevelopmental outcomes with an accuracy of 81% (aOR: 2.59, 95% CI: 1.36–4.95)
Li et al. (31)	62	WBH	ET: 33.5°C Duration: 72 h	Plasma (ELISA)	Elevated Tau protein levels on days 2–3 of hypothermia were associated with death or severe injure on MRI, which may provide the information of infants with a poor outcomes. It was significant for on day 2 of hypothermia (aOR:1.06 for every 1,000 pg/ml increase in Tau, 95% CI: 1.03–1.09, *p* = 0.01) and on day 3 of hypothermia (aOR:1.04 for every 1,000 pg/ml increase in Tau, 95% CI: 1.01–1.06, *p* = 0.07)
Wu et al. (34)	60	SHC	RT: 32°C–34°C Duration: 24 h	Blood and urine (ELISA)	Moderate HIE Before hypothermia treatment: 9.36 ± 1.27 pg/ml After hypothermia treatment: 1 day (8.55 ± 1.32 pg/ml) 3 day (8.21 ± 1.56 pg/ml) 5 day (7.56 ± 1.42 pg/ml) 7 day (7.24 ± 0.95 pg/ml) Severe HIE: Before hypothermia treatment: 14.26 ± 2.85 pg/ml After hypothermia treatment: 1 day (12.87 ± 2.54 pg/ml) 3 day (11.85 ± 2.49 pg/ml) 5 day (10.75 ± 2.49 pg/ml) 7 day (10.49 ± 2.48 pg/ml)
BDNF					
Dietrick et al. (30)	155	NDD	NDD	Plasma or CSF (ELISA)	Moderate to severe cerebral injury: Plasma BDNF level: 387.2 pg/ml (13.6.1–1,006.5 pg/ml); CSF BDNF level: 30.3 pg/ml (30.3–30.3 pg/ml) Cognitive abnormal or death: Plasma BDNF level 9.3 pg/ml (0.5–43.8 pg/ml) Abnormal motor: Plasma BDNF level 414.6 pg/ml (77.0–868.7 pg/ml) Abnormal language: Plasma BDNF level 372.5 pg/ml (77.0–724.9 pg/ml) * In plasma, GFAP level was inversely associated with encephalopathy grade.
UCH-L1					
Chalak et al. (28)	20	WBH	ET: 33.5°C Duration: 72 h	Serum (ELISA)	At 6–24 h after treatment (moderate/severe HIE): 0.6 (0.30–2.2) ng/ml At 48–72 h after treatment: 0.5 (0.3–0.6) ng/ml At 78–96 h after treatment: 0.5 (0.3–0.6) ng/ml
Jiang et al. (35)	31	WBH	RT: 34°C Duration: 72 h	Serum (ELISA)	At 6–12 h after treatment (moderate/severe HIE) 0.7 ± 0.09 ng/ml < before treatment (moderate/severe HIE) 2.3 ± 0.7 ng/ml, *p* < 0.05.
NRGH					
Dietrick et al. (30)	155	NDD	NDD	** **	Moderate to severe cerebral injury: Plasma NRGN level:40 pg/ml (7–403 pg/ml); CSF NRGN level: 7 pg/ml (7–7 pg/ml) Cognitive abnormal or death: Plasma NRGN level 49.9 pg/ml (16.5–243.2 pg/ml) Abnormal motor: Plasma NRGN level 59.4 pg/ml (16.5–314.2 pg/ml) Abnormal language: Plasma NRGN level 38.0 pg/ml (165.5–2.8.2 pg/ml) * In CSF, NRGN level was inversely associated with cognitive, motor and language outcomes. * In plasma, NRGN level was directly associated with encephalopathy grade
Li et al. (31)	62	WBH	ET: 33.5°C Duration: 72 h	Plasma (ELISA)	The relationships between plasma NRGH level and MRI has not significant.

NDD, no detailed description; HIE, hypoxic-ischemic encephalopathy; WBH, whole-body hypothermia; SHC, selective head cooling; NPT, nasopharyngeal temperature; RT, rectal temperature; AFT, anterior fontanelle temperature; ST, skin temperature; ET, esophageal temperature; ELISA, enzyme linked immunosorbent assay; RI, radioimmunity; ACI, automated chemiluminescence immunoassay; CSF, cerebrospinal fluid; NSE, neuron-specific enolase; MBP, myelin basic protein; GFAP, glial fibrillary acidic protein; UCH-L1, ubiquitin carboxyl-terminal hydrolase L1. BDNF, Brain-derived neurotrophic factor. PNF-H, Phosphorylated axonal neurofilament heavy chain; NRGH, Neurogranin; BGP, background pattern.

## NSE

NSE, a dimeric isoenzyme of the glyocytic enzyme enolase and a member of enolase family, with a molecular weight of 78 kDa and biologic half-life of approximately 24 h, which is present in the cytoplasm of neurons and some neuroendocrine cells (31, 36). NSE contains three gene-independent gene fragments (*α*, *β*, *γ*), and it forms five dimeric isoenzymes, namely, αα, ββ, γγ, αγ, and βγ. The *γ* subunit is found in neurons and endocrine tissues, and is also known as NSE (36, 37). The *α*-subunit exists in glia, it is also known as non-NSE. NSE is a key enzyme in the glycolosis pathway, where it catalyzes the conversion of 2-phosphoglyceric acid and phosphoenolpyruvate. NSE not only plays an important catalytic role in glucose metabolism, but it also plays an important role in promoting neuronal differentiation and maturation. Under normal conditions, the concentration of NSE in blood/CSF is lower; the normal range is small and relatively stable. When neurons are damaged, NSE enters CSF or blood, leading to an increased concentration of NSE. Lv et al. (36) reported that NSE had strong specificity and sensitivity and was an early sensitive indicator of neuronal injury. Clinical studies (38, 39) have shown that the serum concentration of NSE was consistent with the severity of neonatal HIE; the higher the NSE concentration, the more serious the neuronal damage. In neonates with severe HIE, the serum concentrations of NSE at 1, 3, and 7 days after birth was 65.79–5.79 ug/L, 60.36–6.66 ug/L, and 52.73–5.67 ug/L, respectively (38). However, Feng et al. (39) showed a concentration of 4.683 ± 4.639 ug/L in neonates with HIE at the 3 days after birth, NSE was positively related to the severity of neonatal HIE, and the concentration in CSF or blood of NSE gradually decreased with improvements in patients' symptoms. Therefore, the changes in the concentration of NSE can be used to evaluate the severity of neonatal HIE or other brain injuries in newborns (40–42). However, there are controversial results. Nagdyman et al. (43) reported that the NSE concentration was not elevated in full-term infants with HIE. Thus, the expression levels of NSE in neonatal HIE still requires further investigation.

There were few studies on the effect of hypothermia for neonatal HIE on the NCS. After selective head cooling to neonatal HIE for 8 h and 72 h, the serum concentrations of NSE was significantly lower than those of a control group (22, 23). Recently, Jia et al. (24) reported that hypothermia administered to neonates with HIE at ≤6 h or ≥6 h after HIE onset led to significant decreases in the serum NSE concentrations in neonates with moderate to severe HIE, it was suggested that hypothermia may reduce serum NSE levels of neonates with moderate to severe HIE. Leon-Lozano et al. (26) showed that HIE infants with seizures, abnormal aEEG, or abnormal MRI had higher significantly CSF-NSE levels compared with compared to those without abnormalities. Thus, we believe that the serum or CSF level of NSE is feasible to assess the effect of hypothermia for neonatal HIE.

## MBP

Brain tissue is composed of gray matter (neuronal cell bodies) and white matter (nerve fiber bundles). After hypoxia-ischemia occurs, gray matter and white matter are affected. White matter is composed of crossing fiber bundles that are interconnected and responsible for signal transmission, and they form a coordinated and unified whole. Myelin is synthesized by glial cells, ensheathing axons with a lipid-rich insulating membrane. It plays a key role in promoting saltatory propagation, preserving the connectivity and function of a healthy nervous system, normal motor, cognitive and sensory functions. In particular, axon myelination is important for the healthy development of brain in newborns and infants (44). Animal models have shown that hypoxemia for neonatal mice with HIE may cause permanent neurological deficits and led to reduced white matter (45). Once HIE occurs, it can cause developmental damage to the brain, including white matter damage and a series of neurological disorders such as cerebral palsy, and long-term cognitive impairment (11).

MBP is a strong basic membrane protein synthesized by oligodendrocytes of CNS and Schwann cells of peripheral nervous system, and it is localized to the cytoplasmic surface, forming the myelin membrane. MBP is an essential structural component of CNS myelin (46), making up approximately 30% of total myelin protein. It binds closely with myelin lipids, maintaining the integrity and stability of the myelin structure (47). Under healthy conditions, MBP easily enters CSF through BBB, and only a small amount of MBP is released into the blood. Once the myelin sheath is damaged (e.g., brain damage or disease), MBP may be released into CSF or blood. Therefore, MBP levels of CSF or blood may reflect the severity of white matter damage, MBP is considered as a specific neurobiochemical marker (48–50). Hu et al. (51) measured serum concentrations of MBP in neonatal HIE, and found that serum MBP concentrations on the 3rd day after birth were 5.12 ± 2.27 ug/L, but the serum MBP concentrations of non-HIE newborns were 1.67 ± 1.42 ug/L. Some scholars have also reported that serum MBP levels were closely related to the degree of brain injury and prognosis (52).

Regarding the effect of hypothermia on MBP in neonatal HIE, Koo et al. (53) reported the changes in MBP levels in neonatal rats (postnatal day 9, using hypothermia) with HIE by immunohistochemical method, showing that hypothermia may reduce the expression of MBP of hippocampus and lateral caudate nuclei, it is suggested that hypothermia may reduce damage of hippocampus and lateral caudate nuclei; this is highly interesting, demonstrating the protective effects of hypothermia for rats with mild brain damage. Another animal model experiment revealed that after sheep with 30 min of cerebral ischemia were treated using hypothermia for 3 h, 48 h, and 72 h, and SMI-312-labeled axons and MBP were quantified in the intragyral white matter, the results showed that ischemia was associated with reduced axonal and myelin area fractions, both ischemia—48 h hypothermia and ischemia—72 h hypothermia may improve axonal area fractions and linearity, suggesting that hypothermia may alleviate post-ischemic axonopathy (54). Histochemical and tissue pathology studies have shown that cerebral ischemia may result in the loss of MBP, and hypothermia was associated with a similar partial improvement in MBP and the number of oligodendrocytes, these results further suggested that the neuroprotective mechanism of hypothermia may be related to reduce microglial activation. However, prolonging hypothermia (extending cooling from 3 to 5 days) did not further improve outcomes, which may be associated with greater numbers of residual microglia (55). Clinically, the changes in expression level of MBP after hypothermia for neonatal HIE are rarely studied. Our previous study showed that the serum MBP levels at 3 days after hypothermia for neonatal HIE (moderate HIE: 5.94 ± 0.68 ug/L; severe HIE: 6.55 ± 0.74 ug/L) were significantly lower than before hypothermia treatment (moderate HIE: 7.02 ± 0.53 ug/L; severe HIE: 8.30 ± 0.73 ug/L), suggesting that hypothermia may reduce the serum MBP levels in neonatal HIE (27). We believe that BMP is a feasible biomarker for the diagnosis of neonatal HIE and evaluation of treatment efficacy.

## S100β

S100β is a member of S100 family that can regulate biological activity via calcium binding. S100 has similar amino acid sequences and structures to other S100 family members, S100α and S100β are the most active in S100 family, and S100β is mainly synthesized by astrocytes of CNS, and can also be found in some neuron subpopulations (36). S100β make up an acidic calcium binding protein with ββ subunits. As a cytokine, S100β has a nutritional function for CNS, and it may be detected in different biofluids (56, 57). S100β is more stable in blood, and is not affected by hemolysis. Normally, the concentration of S100β is low in CSF, blood, and urine. After the brain is damaged, astrocytes may release large amounts of S100β, and it quickly reaches to blood, therefore, S100β is considered to be a sensitive biomarker for early brain injury and the severity damaged disease of CNS. Some scholars believe that S100β is a specific indicator for evaluating the degree of mild brain damage (58). Due to that fact that astrocytes participate in maintaining BBB integrity, S100β concentration in biofluids may be used as a marker of brain and BBB damage (59). Animal experiments by immunohistochemical method have shown that serum S100β levels began to gradually increase 0.5 h after HI and began to decrease at 48 h (59). Normally, the serum levels of S100β in healthy neonates were lower (1.163 ± 0.623 ug/L) at 24 h after birth, the serum level of S100β in neonates with HIE was 3.698 ± 2.761 ug/L (60). Other study reported that serum levels of S100β in neonatal HIE was 16.3 ± 9.5 ug/L (61). Gazzolo et al. (62) found that the urine concentration of S100β in asphyxiated neonates with brain damage was associated with adverse outcomes at 12 months of age, and they determined an S100β concentration cutoff of 0.28 μg/L at first urination, with a sensitivity of 100% and a specificity of 87.3%, to predict the development of abnormal neurological findings on follow-up. Another study of Gazzolo et al. (63) also showed that saliva levels of S100B was significantly higher in asphyxiated full-term newborns with severe neurological outcomes than in those with good neurological outcomes at follow-up and those in healthy controls.

About the effect of hypothermia for neonatal HIE on the S100B, Massaro ae al (64). Reported that the elevated serum S100B levels during hypothermia was showed to be associated with neuroradiographic and clinical evidence of brain madage in neonatal encephalopathy. Alshweki et al. (25) used hypothermia for 31 cases with neonatal HIE, showing that urinary levels of S100β in neonates with unfavorable outcomes was significantly higher than those of neonates with favorable outcomes on the first and second days, and the optimal cutoff of urinary S100β levels on the first day was >1.11 μg/L (sensitivity, 100%; specificity, 60%) for the prediction of neonatal death and <0.66 μg/L (sensitivity 83%; specificity, 70%) for the prediction of a normal neurological examination before discharge. A definitive conclusion concerning the effect of hypothermia for neonatal HIE on S100B cannot currently be drawn from existing studies because of few studies.

## GFAP

GFAP is an acidic protein rich in glutamic acid, and aspartic acid with a molecular mass of 50–52 kD, it is a structural protein composed of intermediate filaments in astrocytes and a specific biomarker of distinguishing astrocytes (36). Astrocytes may synthesize and secrete many components, which including GFAP, S100β, matrix metallopeptidase-9(MMP-9), and neurotrophic factors (36, 65). In the CNS, astrocytes fill spaces between neurons, their processes expands to form terminal end feet, which are attached to adjacent capillary walls or to soft membranes, forming blood-brain barrier and blood–blood barriers. GFAP is a specific biochemical component (or biochemical marker), and it is released into CSF or blood after astrocyte was damaged. Therefore, serum levels of GFAP reflect pathological changes of astrocyte, and may be used to assess BBB damage. Some scholars believe that GFAP is considered as an ideal marker for brain injury. With the use of immunohistochemical method, animal experiments have shown that the number of astrocytes was reduced in newborn piglets with HIE; the cell sizes in the periventricular white matter and the GFAP content of astrocytes were also reduced (66). Previous clinical studies showed that higher serum GFAP levels in neonatal HIE were associated with lower neurobehavioral scores, if serum GFAP levels were ≥0.15 ng/ml, the brain magnetic resonance imaging (MRI) findings will appear in the later stage; the higher serum GFAP levels were associated with higher mortality rates (67). So, GFAP is considered an ideal marker for neonatal brain damage and astrocyte activity (68). However, some studies have obtained different results. Looney et al. (69) reported that cord blood GFAP levels in neonates with perinatal asphyxia/HIE were not higher than those in a healthy control group, and cord blood GFAP levels were not associated with prognosis or outcomes at 36 months. Recently, Dietrick et al. (30) reported that plasma GFAP level was associated with adverse neurodevelopmental outcomes. About the effect of hypothermia for neonatal HIE on GFAP, Chalak et al. (28) reported that after hypothermia for 20 neonates with HIE (17 cases moderate HIE, 3 cases severe HIE), serum GFAP levels were not affected by hypothermia–rewarming. But Jiang et al. (29) reported that hypothermia for neonatal HIE may increase serum levels of GFAP. So, a clear conclusion regarding the effect of hypothermia for neonatal HIE on GFAP cannot be drawn because of inconsistent results among studies.

## Tau protein

Tau protein is a microtubule-associated protein with a molecular mass of 48–67 kDa, and it is a small phosphor protein found in the axonal compartments of neurons (36, 70). Tau protein is bound to axonal microtubules, leading to the formation of axonal microtubule bundles in axon cytoskeletons (71). As a scaffold protein in neurons, Tau protein can promote the stability and assembly of microtubules. Many studies have found higher levels of Tau protein in the CNS in unmyelinated axons and cortical interneurons. After axons were damaged, Tau protein was released from the CNS into CSF and blood, leading to an increase in the serum Tau protein level. Animal models of traumatic brain injury and adult cerebral ischemic disease have shown that serum Tau protein levels were increased, and this was considered a sensitive indicator for early cerebral ischemia (35, 71). Previous literature also reported that serum Tau protein levels on postnatal day 3 were significantly higher in a poor outcome group than those in a good outcome and a control group, and on postnatal day 7, serum Tau protein levels were significantly higher in a poor outcome group than those in a good outcome and control groups again in another study (72), it is suggested that Tau protein and brain injury have a close correlation. Clinical trials have showed that the serum Tau protein level in neonates with severe HIE was significantly higher than that of neonates with moderate HIE, and the serum Tau protein level had an important clinical value for diagnosis and grading of neonatal HIE (30, 31, 33, 73). The findings from our previous study showed that the serum Tau protein levels after hypothermia for neonates with moderate/severe HIE were significantly reduced (32). Recent literature has also shown that hypothermia for moderate/severe HIE could decrease Tau protein levels, and indicated that Tau protein was a useful indicator for early diagnosis of HIE (34). Decreased Tau proteins expression after hypothermia could be associated with the stabilization plasma membranes of neuron, reducing lipid peroxidation, regulating phosphorylation of Tau protein, reducing microtubule rupture, maintaining normal morphology of neuron and axon transport of neurons, and reducing brain injury (74). We believe that Tau protein is a promising marker for predicting neonatal brain damage.

## UCHL1

UCHL1, also known as protein gene product 9.5, is an important member of the ubiquitin–protease system. A cysteine hydrolase consisting of 233 amino acids with a molecular mass of 24,800, it is estimated to represent 1%–2% of the total brain proteins. UCHL1 is widely distributed in the brain tissue of vertebrates and in neuronal cytoplasm in humans, it has been used as a marker for neuron health (75–77). Using an animal model of foals with HIE, Ringger et al. (78) reported that median UCHL1 levels (6.57 ng/ml; 2.35–11.90 ng/ml) in foals with HIE were significantly higher than those of healthy controls (2.52 ng/ml; 1.4–4.01 ng/ml), the sensitivity and specificity of UCHL1 (>4.01 ng/ml) for diagnosis of HIE were respectively 70% (51%–84%) and 94% (72%–99%), and UCHL1 levels were higher in gray matter than in white matter, demonstrating that UCHL1 is a marker of brain injury in foals with HIE. Recently, it was shown that cord blood UCHL1 levels were higher in neonatal HIE, and there was a close correlation between UCHL1 levels and brain injury in neonatal HIE (13, 29). Serum UCH-L1 levels were shown to be valuable to diagnose neonatal HIE. Due to limitations in sample size of these previous studies, further studies are necessary with larger sample sizes.

Regarding the effect of hypothermia for neonatal HIE on UCH1, Chaliak et al. (28) first reported serum UCHL1 levels in neonatal HIE after hypothermia therapy. Then, Jiang et al. (29) reported that serum UCH-L1 levels were significantly lower in neonates with moderate/severe HIE after hypothermia treatment (for 6–12 h) than those in neonates with mild HIE (conventional treatment) before treatment. Similar results were also obtained in our previous study (79), suggesting that UCH-L1 may be used as a promising biomarker of brain damage in neonates with HIE.

## BDNF

BDNF was first discovered in pig brains by Barde et al. in 1982. It is a neurotrophic factor; the molecule monomer BDNF is a mature polypeptide composed of 119 amino acid residues. The isoelectric point of is 9.99, with a relative molecular mass of 3.5–103 (36, 65). It is an alkaline protein and mainly consisting of *β* folding and irregular curl secondary structures, with three disulfide bonds. Myriad experimental data have shown that BDNF is widely expressed in the nervous system, endocrine system, bone and cartilage, but it is mainly expressed in neurons and astrocytes in the CNS, and the BDNF content of hippocampus and cortex are the highest, BDNF's functions include promoting growth, differentiation, regeneration, and repair of neurons (80, 81). It has been shown that the plasma BDNF level increased after neonatal HIE, which was associated with adverse neurodevelopmental outcomes (30). With the use of immunohistochemistry method, Diaz et al. (82) found that the BDNF levels in the ipsilateral forebrain in mice with hypoxic–ischemic injury on postnatal day 10 were 1.7- to 2-fold higher than those in the sham-treatment forebrain, and hypothermia did not prevent this increase. A clinical study (81) also showed that the BDNF level significantly increased at 72 h of life after hypothermia compared to its level at delivery among cases. These findings suggest that hypothermia has a function of enhancing endogenous BDNF. However, the mechanism should be further studied.

## PNF-H

PNF-H is a member of the neurofilament protein family, with a molecular weight of 200,000, and a type VI intermediate filament protein with a helical structure. It can bind keratin to form a dense structure; the C terminal of neurofilament protein H (NF-H) contains 20 polypeptide chains (6–8 amino acids each) (83). Each amino acid polypeptide chain contains lysine–serine–proline groups. Different phosphorylation forms of NF-H exist in different parts of neurons, and highly phosphorylated NF-H (PNF-H) exists only in axons, where it promotes axonal growth and plays an important role in signal transmission (25, 84). Upon neuronal injury, the expression of PNF-H is increased, and PNF-H levels were positively correlated with the degree of brain injury (85). Therefore, PNF-H in the blood has been considered a marker of brain injury. Encephalopathy or spinal axonal injury can lead to elevated of PNF-H levels in biofluids (e.g., encephalitis, encephalomyelitis, hydrocephalus, subarachnoid hemorrhage, spinal muscular atrophy, multiple sclerosis, stroke) (86–90). In a neonatal foal model of HIE, serum PNF-H levels were significantly higher in the HIE group than in the healthy control group, indicating that serum PNF-H was a potential marker of neonatal brain injury. PNF-H levels were higher in white matter than in gray matter (33). However, Patil et al. (21) reported that no significant difference between serum PNF-H levels of a neonatal HIE group and a healthy neonate group. The effect of hypothermia for neonatal HIE on PNF-H is not yet full understood, which still needs to be further studied.

## Ng

Ng is discovered in 1990, which is a calmodulin-binding protein with a molecular weight of 7.5 kD and composed of 78 amino acids (91). It has been reported that Ng mainly distributed in the telencephalon, specifically located in the cell bodies and dendritic processes of neurons of the cerebral cortex, hippocampus, striatum, and a few other discreet areas (92). Ng is a small protein usually expressed in granule-like structures in pyramidal cells of the hippocampus and cortex, so it is also known as “neurogranulin”. Currently, Ng is proved to be involved in synaptic plasticity, synaptic regeneration, and long-term potentiation mediated by the calcium- and calmodulin-signaling pathways (93, 94). As one of the brain-specific proteins, many scholars have found that the expression level of Ng in the serum or CSF is elevated in many CNS diseases, such as traumatic brain injury (95), acute ischemic stroke (96), schizophrenia and depression (91, 97). Therefore, it is suggested that Ng can be used as a marker of central neuronal injury. Recently, few studies have investigated the plasma and CSF levels of Ng in neonatal encephalopathy. Dietrick et al. (30) studied 155 patients with neonatal encephalopathy, and found that the plasma level of Ng was closely related to the grading of neonatal encephalopathy, while the CSF level of Ng was related to the prognosis of neonatal encephalopathy such as cognitive, motor and language abnormalities. Nevertheless, Li et al. (31) studied 62 neonates with moderate-severe HIE, and found no correlation between Ng levels and brain damage revealed by MRI. The reasons for this difference is not clear, we believe that the association of Ng with neonatal HIE or neonatal encephalopathy still needs to be further investigated.

The limitation of this article is that this is a literature review, which is less systematic and lacks the rigorous systematic methodology of systematic reviews. Additionally, only a small number of published studies reported the effect of hypothermia for neonatal HIE on CNSPs, which limits the amount of information gathered or extracted from each study included in the sample. And a clear conclusion usually cannot be drawn because some studies lack uniformity in diagnostic criteria and sample collection time. Despite these limitations, this article includes latest literature, provides a comprehensive understanding of the classification, origin, biochemical composition, and physiological function of CNSPs, as well as changes in their expression levels after hypothermia for neonatal HIE, thus improving the awareness of CNSPs among pediatricians.

## Conclusions

The use of CNSPs as specific biochemical signals in biofluids comes with a good ease of use and has demonstrated strong advantages in the early diagnosis and evaluation of neonatal HIE. Further research is needed to explore the mechanisms behind the effects of hypothermia on these biochemical signals.
